# Fabrication and Evaluation of Screen-Printed Electrodes on Chitosan Films for Cardiac Patch Applications with In Vitro and In Vivo Evaluation

**DOI:** 10.3390/polym17152088

**Published:** 2025-07-30

**Authors:** Yu-Hsin Lin, Yong-Ji Chen, Jen-Tsai Liu, Ching-Shu Yen, Yi-Zhen Lin, Xiu-Wei Zhou, Shu-Ying Chen, Jhe-Lun Hu, Chi-Hsiang Wu, Ching-Jung Chen, Pei-Leun Kang, Shwu-Jen Chang

**Affiliations:** 1 Department of Cardiovascular Surgery, Pingtung Veterans General Hospital, Pingtung City 900053, Taiwan; p038@ptvgh.gov.tw; 2 Department of Biomedical Engineering, I-Shou University, Kaohsiung City 824005, Taiwan; isu10901004d@cloud.isu.edu.tw (Y.-J.C.); isu10050004m@cloud.isu.edu.tw (C.-S.Y.); isu9950059a@cloud.isu.edu.tw (Y.-Z.L.); isu9850096a@cloud.isu.edu.tw (X.-W.Z.); shufen0831@gmail.com (S.-Y.C.); isu11350003m@cloud.isu.edu.tw (J.-L.H.); isu11250003m@cloud.isu.edu.tw (C.-H.W.); 3 Research Center for Materials Science and Opti-Electronic Technology, College of Materials Science and Opto-Electronic Technology, University of Chinese Academy of Sciences, Beijing 100049, China; jtliu@ucas.ac.cn; 4 Research Center for Materials Science and Opti-Electronic Technology, School of Optoelectronics, University of Chinese Academy of Sciences, Beijing 100049, China

**Keywords:** chitosan, screen-printed electrodes (SPEs), myocardial infarction, electrical stimulation, cardiac regeneration

## Abstract

Myocardial infarction (MI) remains one of the most common cardiovascular diseases and a leading cause of morbidity and mortality worldwide. In recent years, natural polymeric patches have attracted increasing attention as a promising therapeutic platform for myocardial tissue repair. This study explored the fabrication and evaluation of screen-printed electrodes (SPEs) on chitosan film as a novel platform for cardiac patch applications. Chitosan is a biodegradable and biocompatible natural polymer that provides an ideal substrate for SPEs, providing mechanical stability and promoting cell adhesion. Silver ink was employed to enhance electrochemical performance, and the electrodes exhibited strong adhesion and structural integrity under wet conditions. Mechanical testing and swelling ratio analysis were conducted to assess the patch’s physical robustness and aqueous stability. Silver ink was employed to enhance electrochemical performance, which was evaluated using cyclic voltammetry. In vitro, electrical stimulation through the chitosan–SPE patch significantly increased the expression of cardiac-specific genes (GATA-4, β-MHC, troponin I) in bone marrow mesenchymal stem cells (BMSCs), indicating early cardiogenic differentiation potential. In vivo, the implantation of the chitosan–SPE patch in a rat MI model demonstrated good tissue integration, preserved myocardial structure, and enhanced ventricular wall thickness, indicating that the patch has the potential to serve as a functional cardiac scaffold. These findings support the feasibility of screen-printed electrodes fabricated on chitosan film substrates as a cost-effective and scalable platform for cardiac repair, offering a foundation for future applications in cardiac tissue engineering.

## 1. Introduction

Myocardial infarction (MI) remains one of the leading causes of death and long-term morbidity worldwide. The irreversible loss of cardiomyocytes after MI leads to fibrotic scarring, myocardial wall thinning, and a progressive decline in cardiac function [1]. Despite advances in medical and surgical interventions, current treatments remain insufficient to regenerate the damaged myocardium, resulting in limited recovery and an increased risk of heart failure [2,3]. As the incidence of MI and its associated long-term complications continues to rise, there is a growing need for novel therapeutic strategies that can effectively regenerate damaged cardiac tissue and restore cardiac function. Therefore, there is growing interest in bioengineered cardiac patches that not only provide mechanical support but also foster a regenerative microenvironment that promotes cell survival and tissue integration [4]. These patches have the potential to reduce fibrosis, enhance tissue remodeling, and restore myocardial function. Among the various strategies for cardiac tissue repair, the use of biocompatible materials, particularly natural polymers such as chitosan, has shown great promise [5,6].

Chitosan is a biodegradable polysaccharide derived from chitin that has attracted much attention for its excellent mechanical properties, cytocompatibility, and its ability to promote tissue integration [6,7,8]. It has been extensively explored for applications including drug delivery systems, wound-healing dressings, antimicrobial coatings, and tissue engineering scaffolds. The ability of chitosan to promote cell adhesion, modulate immune responses, and support extracellular matrix formation makes it a valuable material across different tissue repair and regenerative medicine strategies [9,10,11]. Chitosan-based scaffolds have been reported to support cell attachment, reduce inflammation, and promote structural repair of cardiac tissue [12,13,14]. However, structural support alone is often insufficient for complete cardiac regeneration, particularly given the heart’s reliance on tightly regulated electrical and mechanical activity. To address this, researchers have investigated strategies that not only restore tissue architecture but also facilitate physiological functions necessary for synchronized contraction and integration with the host myocardium. Among these, the incorporation of conductive properties into cardiac scaffolds has attracted significant interest due to their potential to bridge biological and bioelectrical functions in damaged cardiac tissue.

The electrophysiological properties of the heart, including the generation and propagation of action potentials, are crucial for maintaining synchronized cardiac contractions. These properties provide the rationale for the application of electrical stimulation in regenerative medicine, which has been shown to promote stem cell differentiation and enhance tissue repair [15,16]. By mimicking the intrinsic electrical activity of the heart, electrical stimulation can improve the alignment, maturation, and integration of newly differentiated cardiomyocytes, processes that are essential for effective cardiac tissue regeneration [17]. Consequently, electrical stimulation has emerged as a promising therapeutic approach for myocardial repair, particularly in the context of myocardial infarction (MI) [18,19].

Chitosan has been investigated as a substrate for screen-printed electrodes (SPEs), which are used to create conductive interfaces for various biomedical applications, including cardiac tissue repair. Previous studies, such as Lin et al. (2018), demonstrated that chitosan films possess favorable mechanical characteristics, making them suitable for use as substrates in electrode fabrication [20]. Screen printing is a widely used cost-effective, and adaptable method for producing electrodes, particularly for regenerative medicine applications [21]. This technique involves depositing a conductive ink onto a substrate through a mesh screen, enabling the creation of scalable and precise electrode patterns [22]. The high-resolution electrodes produced using this method are essential to ensure optimal electrochemical performance in biological applications. Furthermore, the flexibility of screen printing enables the use of a variety of biopolymer substrates, including chitosan, enhancing its potential for myocardial regeneration [23]. The scalability and design flexibility of this method, coupled with its capacity to incorporate electrical stimulation, highlight its potential as a promising tool for myocardial regeneration.

In this study, we propose a chitosan-based screen-printed electrode (SPE) patch as a multifunctional platform for myocardial regeneration. We hypothesize that combining biocompatible chitosan films with scalable screen printing techniques can yield conductive patches that support both mechanical integration and electrochemical interfacing with host myocardium. To test this hypothesis, we systematically investigate the dimensional stability, mechanical strength, electrode adhesion, and electrochemical performance of the chitosan–SPE constructs. Furthermore, we assess their in vivo compatibility and regenerative capacity using a myocardial infarction (MI) model through histological evaluation. While the broader role of electrical stimulation and stem cell differentiation in cardiac repair is acknowledged, the primary objective of this work is to validate the material properties and myocardial integration potential of chitosan–SPE patches. Collectively, our findings aim to establish a bioelectronic interface capable of supporting structural repair and offering a foundation for future developments in cardiac electroceutical therapies.

## 2. Materials and Methods

### 2.1. Reagents

Chitosan of two different molecular weights, 70 kDa and 300 kDa, and ≥75% deacetylated was purchased from Sigma (Sigma-Aldrich, St. Louis, MO, USA). Sodium hydroxide (NaOH, ACS reagent, ≥97.0%, Sigma-Aldrich, St. Louis, MO, USA), acetic acid (99.7%, Mallinckrodt, Staines-upon-Thames, UK), phosphate-buffered saline (PBS, pH 7.4), carbon ink (SC-1010, ITK), and silver ink (NT-6307-2, PERM TOP) were used in film preparation and electrode fabrication. All other chemicals used in this study were of reagent grade.

### 2.2. Preparation of Chitosan Films

Chitosan powder was dissolved in 0.1 N acetic acid solution to prepare 2% (*w*/*v*) chitosan solutions. The solutions were cast into Petri dishes and dried overnight in an oven at 40 °C to obtain uniform thin films. Gelation was induced by immersing the films in 1 N NaOH for variable periods at room temperature. After gelation, the films were thoroughly washed with distilled water to remove residual reagents and subsequently air-dried under ambient conditions. The resulting films were stored in a desiccator maintained at room temperature (approximately 22–25 °C) with controlled relative humidity between 20% and 30% prior to use. Prior to use in both in vitro and in vivo experiments, the chitosan–SPE patches were sterilized by continuous ultraviolet (UV) irradiation for 24 h under aseptic conditions in a laminar flow cabinet.

### 2.3. Characterization of Chitosan Films

#### 2.3.1. Swelling Ratio Determination

To evaluate the swelling behavior of the chitosan films, their surface area was measured before and after immersion in phosphate-buffered saline (PBS, 7.4). The films were immersed in 10 mL of PBS solution for 60 min. After this period, the surface area of the swollen films was recorded. The swelling ratio was calculated using the following formula:

$$Swelling\;ratio=\frac{A_{w}-A_{d}}{A_{d}}$$

where *A_w_* is the surface area of the swollen film, and *A_d_* is the surface area of the dry film. The surface areas were calculated from top-view photographs using ImageJ software, version 1.53t (National Institutes of Health, NIH USA) by outlining the film boundaries and performing pixel-based area quantification. The results are expressed as the average ± standard deviation (SD).

#### 2.3.2. Mechanical Strength Test

To evaluate the mechanical strength of the films, rectangular strips (1 × 6 cm^2^) were soaked in pH 7.4 PBS solution for 24 h to simulate physiological conditions. The films were then subjected to a tensile test at a constant rate of 10 mm/min using an MTS testing system (Model H1KS; Tinius Olsen, Horsham, PA, USA) equipped with a 50 N load cell. Stress–strain curves were recorded for analysis.

### 2.4. Electrode Fabrication

Chitosan films (70 kDa and 300 kDa) were used as substrates for screen printing (Model NSP-1A, YULISHIH INDUSTRIAL Co., Ltd., New Taipei City, Taiwan). Carbon ink (SC-1010, ITK, Tainan City, Taiwan) and silver ink (NT-6307-2, PERM TOP, Taoyuan City, Taiwan) were employed to fabricate the electrodes. The screen printing process was carried out in sequence, followed by drying the carbon electrodes at 60 °C for 30 min and the silver electrodes at 120 °C for 60 min to ensure complete curing and adhesion to the chitosan substrate.

### 2.5. Characterization of Electrode Films

#### 2.5.1. Adhesion Test of SPE

To assess the adhesion of carbon and silver inks on chitosan, the cross-cut method was employed. The printed chitosan films were cut into a 5 × 5 grid (25 squares) within a 2 × 2 cm^2^ area using a precision blade. A pressure-sensitive adhesive tape was applied to the cut surface, removed, and the adhesion performance was evaluated according to ASTM D3359-95 standard [24]. Polycarbonate (PC) films were used as the control group for comparison. The adhesion quality was assessed using an evaluation scale ranging from 5B (best) to 0B (poorest).

#### 2.5.2. Cyclic Voltammetry

Electrochemical properties were assessed using cyclic voltammetry to evaluate the charge transfer characteristics of the screen-printed electrodes. All electrochemical measurements were performed using a three-electrode system with the screen-printed electrode as the working electrode, a platinum wire as the counter electrode, and a Ag/AgCl electrode as the reference. The electrodes were tested in a 0.1 mM potassium ferricyanide solution prepared in PBS. Measurements were conducted at a scan rate of 100 mV/s for 20 consecutive cycles using a computer-controlled potentiostat.

### 2.6. Cell Culture and In Vitro Electrical Stimulation Assays

To evaluate the electroactive functionality of the screen-printed electrodes, in vitro tests were conducted using mesenchymal stem cells (MSCs) as a model to assess the biological response to electrical stimulation. These assays aimed to determine whether electrical cues delivered via the chitosan-based electrodes could induce cardiac-specific gene expression in MSCs.

#### 2.6.1. Isolation and Culture of Mesenchymal Stem Cells (MSCs)

All procedures conformed to the guidelines of the Institute of Animal Care and Use Committee of I-Shou University (IACUC-ISU-103022). Mesenchymal stem cells (MSCs) were isolated from the femurs of 3-week-old Sprague–Dawley rats. Briefly, after aseptic preparation, tibias and femurs were isolated, and the soft tissues were removed. The marrow cavities were rinsed with phosphate-buffered saline (PBS), and bone marrow was flushed out using a syringe filled with low-glucose Dulbecco’s Modified Eagle Medium (LG-DMEM) supplemented with 10% fetal bovine serum (FBS) and 200 U/mL penicillin/streptomycin. The resulting cell suspension was filtered and seeded into culture plates containing fresh medium. The cells were incubated at 37 °C in a humidified atmosphere with 5% CO_2_. MSCs were isolated based on their adherence properties and subcultured in LG-DMEM. Cells from the second passage (P2) were used for further experiments.

#### 2.6.2. Electrical Stimulation and Chemical Induction

**Figure 1:**
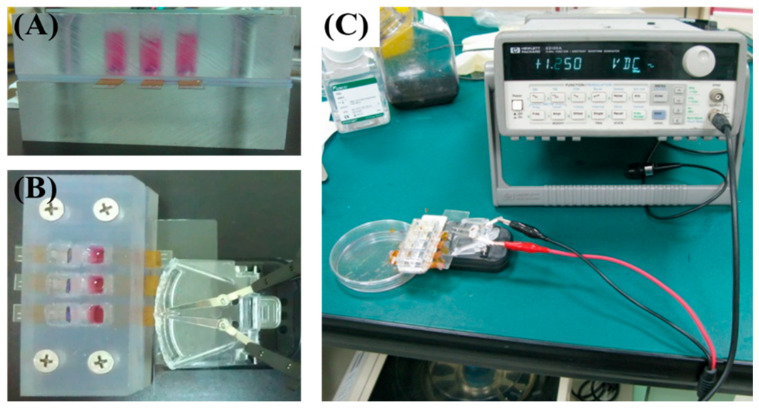
Electrical stimulation setup. (**A**) Photograph of the cell culture chamber. (**B**,**C**) Photographs showing the signal generator connected to the chitosan–SPE integrated within the culture chamber.

**Figure 2:**
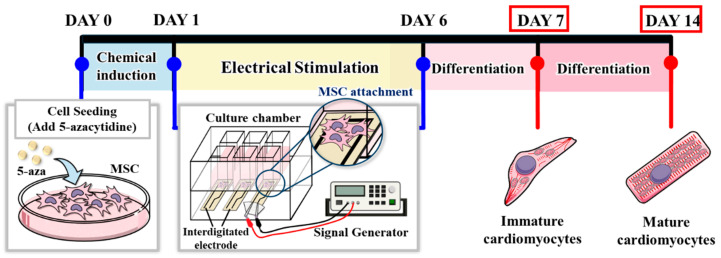
Schematic timeline of chemical induction and electrical stimulation protocol.

The electrical stimulation system is illustrated in Figure 1. The chitosan–SPE was integrated into the cell culture platform and connected to a signal generator. MSCs were seeded at a density of 1 × 10^4^ cells per well. For the chemical induction group, cells were pretreated with 5-azacytidine (5-Aza) for 24 h. Electrical stimulation was applied following chemical induction using square pulses of 2 ms duration at 1 Hz and an amplitude of 100 mV. The stimulation was administered daily until day 6, as shown in Figure 2. Cells were harvested on days 7 and 14 for cardiac-specific gene expression analysis. The experimental groups and treatment conditions are summarized in Table 1.

#### 2.6.3. Quantitative Real-Time Polymerase Chain Reaction (qRT-PCR)

The expression of cardiac-specific genes, including Troponin I, β-MHC, and GATA-4, was evaluated by RT-qPCR. Total RNA was extracted from MSCs using the RNeasy^®^ Plus Micro Kit (Qiagen, Hilden, Germany), and cDNA synthesis was performed using the iScript™ cDNA Synthesis Kit (Bio-Rad, Hercules, CA, USA). SYBR Green Master Mix, gene-specific primers, and synthesized cDNA were used for the RT-PCR reactions, which were carried out on the StepOne™ Real-Time PCR System (Applied Biosystems, Foster city, CA, USA). The primer sequences for the target genes are listed in Table 2, with Arbp serving as the housekeeping gene. The relative expression levels were calculated using the 2^−ΔΔCT^ method.

### 2.7. In Vivo Evaluation of Screen-Printed Cardiac Patches for Myocardial Repair

#### 2.7.1. Myocardial Infarction Model and Cardiac Patch Implantation

All procedures conformed to the guidelines of the Institute of Animal Care and Use Committee of I-Shou University (IACUC-ISU-103022). Eight-week-old female SD rats were anesthetized with an intramuscular injection of 20–40 mg/kg Zoletil 50, followed by endotracheal intubation and mechanical ventilation. A left thoracotomy was performed, and the middle portion of the left anterior descending coronary artery (LAD) was ligated using a 7-0 polypropylene suture. Three weeks post infarction, the chitosan film with screen-printed electrodes was sutured onto the infarcted area as a cardiac patch. A schematic representation of the MI induction procedure and cardiac patch implantation is shown in Figure S1.

#### 2.7.2. Histological Assessment

Hearts were harvested four weeks post implantation of the cardiac patch. In the MI group, hearts were collected at the fourth week post myocardial infarction as a control. The excised hearts were washed several times with PBS solution, fixed in 10% formaldehyde, and sectioned perpendicular to the myocardial defect (MI region). The tissue was then embedded in paraffin blocks and stained with hematoxylin and eosin (H&E) for histological evaluation.

### 2.8. Statistics

All data are presented as mean ± standard deviation (SD). Statistical analyses were performed using a one-way analysis of variance (ANOVA) with Tukey’s post hoc test. Experiments were conducted with *n* = 3–4 per group unless otherwise specified. A *p*-value of <0.05 was considered statistically significant. Statistical analyses were performed with GraphPad Prism 8.0 (GraphPad Software, Inc., San Diego, CA, USA).

## 3. Results

### 3.1. Characteristics of Chitosan Films

Scanning electron microscopy (SEM) revealed that both 70 kDa and 300 kDa chitosan films exhibited smooth and uniform surfaces (Figure S1), confirming the effectiveness of the film fabrication process and the suitability of the resulting substrates for screen printing applications.

**Figure 3:**
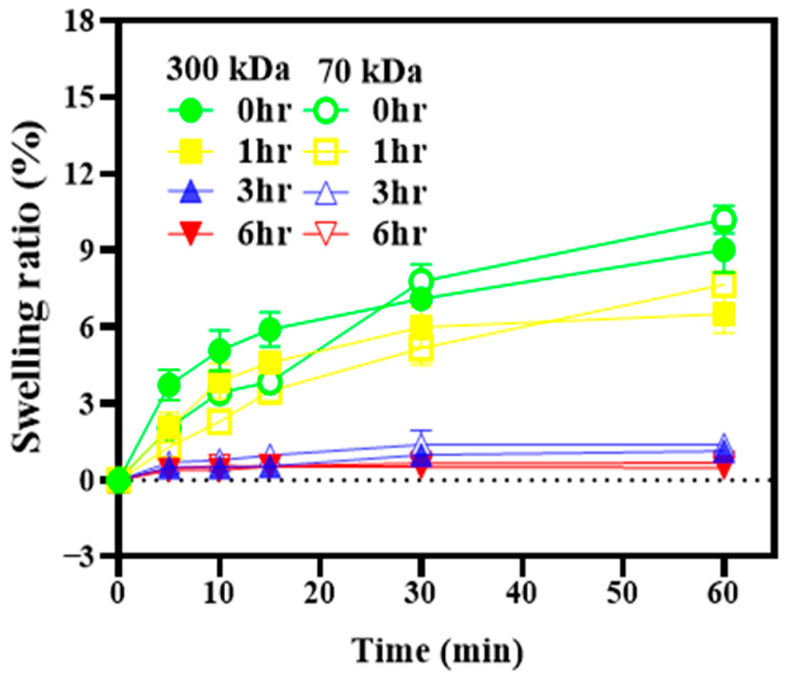
Swelling ratios of chitosan films with different molecular weights and crosslinking durations. The films were immersed in phosphate-buffered saline (PBS) at room temperature, and changes in surface area were used to calculate the swelling ratios.

Chitosan is a hydrophilic polymer that absorbs water and tends to swell in aqueous environments, which may compromise the dimensional integrity of printed structures. To evaluate dimensional stability, the surface area of the films was calculated from measured length and width before and after immersion in phosphate-buffered saline (PBS). As shown in Figure 3, both 70 kDa and 300 kDa chitosan films crosslinked for 3 h or longer exhibited swelling ratios below 4%, with virtually no change in surface area. Based on these results, a 3 h crosslinking duration was selected as the optimal condition, effectively minimizing swelling while avoiding excessive processing time.

**Figure 4:**
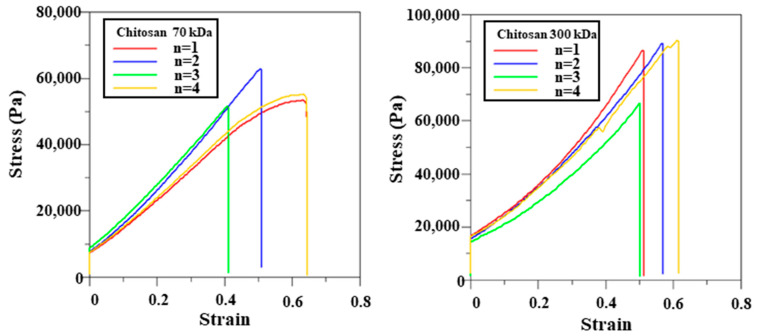
Mechanical properties of chitosan films with different molecular weights.

The mechanical properties of the films were further assessed using tensile testing. Stress–strain curves are shown in Figure 4, and the corresponding mechanical parameters are summarized in Table 3. The 300 kDa chitosan film crosslinked for 3 h demonstrated superior mechanical performance, with higher tensile strength (83.65 ± 11.34 kPa) and Young’s modulus (152.57 ± 18.53 kPa) compared to the 70 kDa film (56.27 ± 4.95 kPa and 105.39 ± 23.99 kPa, respectively). Both film types exhibited comparable elongation at break, with tensile strains around 0.55 and displacement values of approximately 21.9 mm. These findings indicate that the 300 kDa chitosan film offers enhanced mechanical strength and stiffness while maintaining flexibility, making it a favorable substrate for screen-printed electrode fabrication.

### 3.2. Adhesion Test of Screen-Printed Electrodes (SPEs)

**Figure 5:**
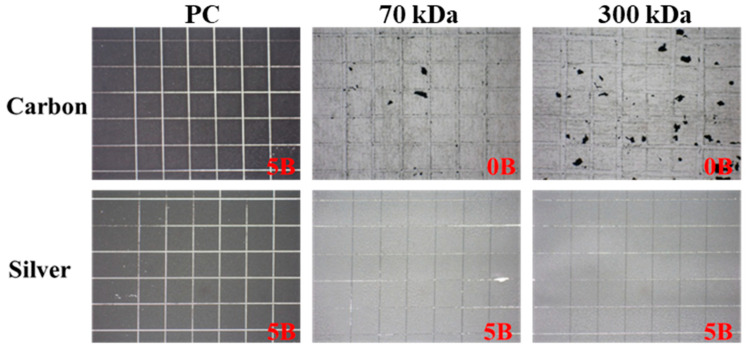
Adhesion performance of carbon and silver inks on chitosan and PC films under wet conditions.

Portable sensors fabricated using screen printing techniques are commonly employed in the biomedical field; however, limited research has investigated the use of natural polymers as substrates in such applications. This study evaluated the adhesion properties of both carbon and silver inks on chitosan and polycarbonate (PC) films, employing the cross-cut method. Since natural polymer substrates such as chitosan are likely to be used in biomedical environments that involve contact with tissue or bodily fluids, ink adhesion was assessed under wet conditions to better reflect environments relevant to biomedical applications (Figure 5). Under these conditions, carbon ink completely lost adhesion to the chitosan films, receiving a rating of 0B for both molecular weights. In contrast, silver ink maintained strong adhesion, with a 5B rating across all film types, including both chitosan and PC. These results suggest that silver ink is more suitable for screen printing on chitosan films, particularly for biomedical applications where exposure to aqueous environments is anticipated. Based on these findings, silver-based screen-printed electrodes were selected for all subsequent experiments.

### 3.3. Cyclic Voltammetry

**Figure 6:**
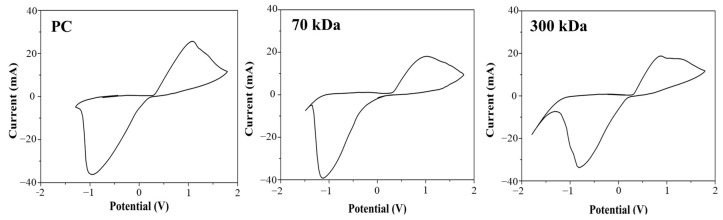
Electrochemical CV responses of the Ag electrode on different substrates, evaluated in 0.1 mM K_3_[Fe(CN)_6_], in buffer, pH 7.2.

Cyclic voltammetry (CV) was employed to evaluate the electrochemical performance of silver electrodes printed on chitosan films with different molecular weights (70 kDa and 300 kDa). As shown in Figure 6, the electrodes exhibited well-defined redox peaks with narrow peak-to-peak separations and clear redox activity, suggesting favorable electron transfer behavior, despite some asymmetry in peak currents. No significant differences were observed between the two chitosan substrates, indicating that the molecular weight of the chitosan did not notably affect the redox behavior of the silver electrodes.

Based on these assessments, the 300 kDa chitosan film crosslinked for 3 h was identified as the optimal substrate for screen-printed silver electrodes. It offered a favorable combination of dimensional stability, mechanical integrity, and electrochemical performance. To further investigate the biological effects of the chitosan-based electrode platform, gene expression analysis was conducted to evaluate cellular responses at the molecular level.

### 3.4. Gene Expression of MSCs Induced by Electrical Stimulation

**Figure 7:**
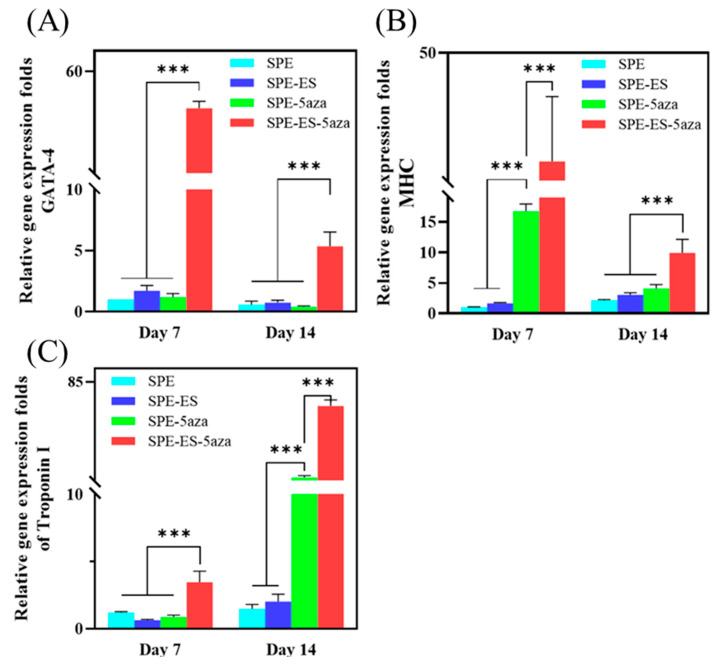
Quantitative real-time PCR analysis of cardiac-specific gene expression (**A**) GATA-4, (**B**) β-MHC, and (**C**) Troponin I in MSCs cultured under different treatment conditions on day 7 and day 14. Experimental groups included SPE (cells cultured on screen-printed electrodes), SPE-5Aza (SPE + 24 h pre-treatment with 5-azacytidine), SPE-ES (SPE + electrical stimulation), and SPE-ES-5Aza (SPE + 5-azacytidine + electrical stimulation). Gene expression levels were normalized to Arbp (acidic ribosomal phosphoprotein P0) as the housekeeping gene and are expressed as fold changes relative to the SPE group. Data are presented as mean ± SD (*n* = 3). *** *p* < 0.001 vs. SPE group.

To evaluate the biological functionality of the chitosan–SPE platform, an electrical stimulation system was integrated into a custom-designed cell culture chamber (Figure 1A–C) to deliver controlled electrical signals through silver-based screen-printed electrodes to mesenchymal stem cells (MSCs) cultured on chitosan films during cardiac induction. As shown in Figure 7A, qRT-PCR analysis of the early cardiac transcription factor GATA-4 revealed that the group receiving both electrical stimulation and 5-azacytidine (5-Aza) treatment (SPE-ES-5Aza) exhibited significantly higher expression on day 7 compared to day 14 and to all other groups. Similarly, Figure 7B shows that β-MHC, another early cardiac marker, was markedly elevated in the SPE-ES-5Aza group on both day 7 and 14, with higher expression observed on day 7. Figure 7C presents the expression of Troponin I, a late-stage cardiac marker, which was significantly upregulated in the SPE-ES-5Aza group at both time points, with a peak at day 14. These results indicate that the combination of electrical stimulation and 5-Aza treatment synergistically enhances the cardiac differentiation of MSCs, as evidenced by the sequential, time-dependent upregulation of early and late cardiac markers.

### 3.5. Cardiac Patch Implantation in the Injured Myocardium

The successful establishment of the myocardial infarction (MI) model was confirmed by gross morphological examination. Three weeks after LAD ligation, the infarcted region appeared clearly demarcated and pale in comparison to healthy myocardium, indicative of fibrotic tissue formation (Figure S3B vs. Figure S3A). This confirmation validated the induction of myocardial injury and established a suitable platform for subsequent cardiac patch implantation.

**Figure 8:**
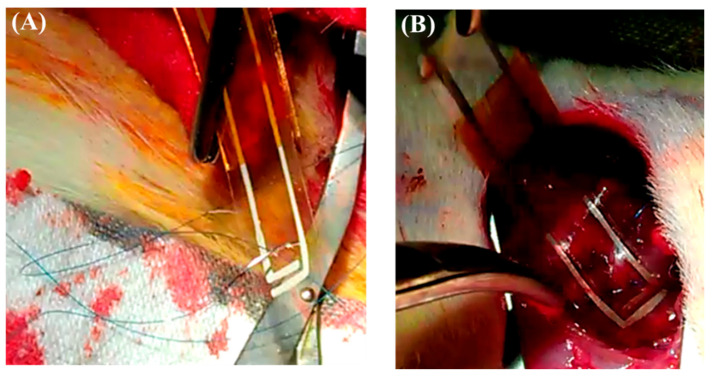
The chitosan patch was sutured onto the infarcted zone of the rat heart to secure its position. (**A**) The patch exhibited sufficient mechanical strength and flexibility, enabling it to be sutured onto the epicardial surface without tearing the conductive layer. (**B**) The patch remained securely attached to the infarcted region after implantation, conforming well to the heart surface and maintaining positional stability.

Both mechanical strength and flexibility are essential characteristics for a cardiac patch, ensuring conformity to the heart’s surface and resilience during surgical manipulation. Materials lacking sufficient mechanical robustness may tear during suturing, compromising implant stability and efficacy. Following MI confirmation, the chitosan–SPE patch was sutured onto the epicardial surface of the infarcted area (Figure S2), and it exhibited adequate strength to withstand suturing without tearing the conductive layer (Figure 8A). The patch also adhered securely to the heart tissue (Figure 8B), demonstrating its suitability for cardiac repair applications.

**Figure 9:**
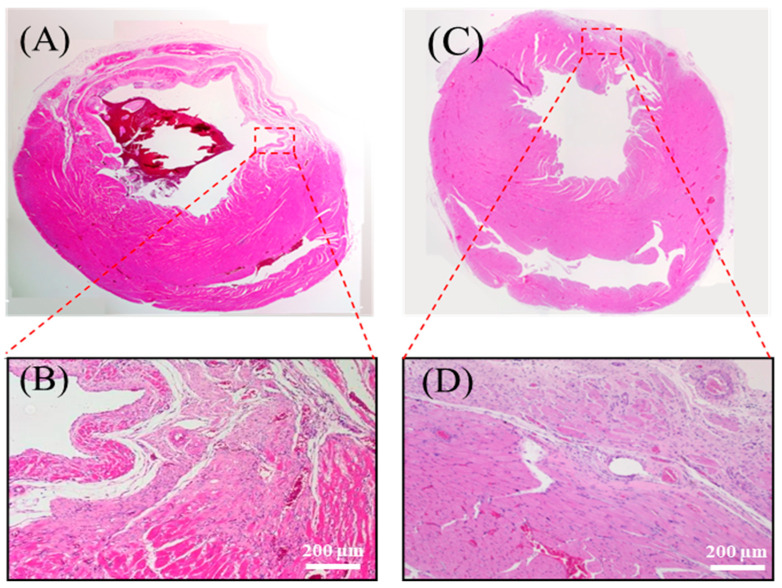
Histological analysis of myocardial tissue post implantation. (**A**) Full heart section from the MI group. (**B**) Magnified view of the infarcted region in the MI group. (**C**) Full heart section from the chitosan patch with the SPE group. (**D**) Magnified view of the infarcted region in the chitosan patch with the SPE group. The patch used in this study was fabricated with 300 kDa chitosan, and the images were obtained from in vivo myocardial tissue sections following implantation.

At four weeks post implantation, histological analysis revealed distinct pathological differences between the control and experimental groups. The untreated myocardial infarction (MI) group exhibited classic features of adverse cardiac remodeling, including ventricular wall thinning, regional dilation (Figure 9A), disorganized myocardial fibers, and infiltration of adipose and inflammatory cells (Figure 9B).

Conversely, in rats implanted with the chitosan–SPE patch, the infarcted region showed signs of structural preservation. The wall was markedly thicker, the fibrotic area was reduced (Figure 9C), and inflammatory infiltration was attenuated. Some myocardial fiber alignment was observed in the repaired zone (Figure 9D), suggesting that the patch promoted a more favorable healing microenvironment. Importantly, the chitosan-based patch appeared to have fully degraded by the four-week endpoint, consistent with its role as a biodegradable temporary scaffold that supports tissue recovery without eliciting noticeable immune responses.

## 4. Discussion

This study explored the potential of chitosan-based screen-printed electrode (SPE) patches for cardiac tissue engineering, emphasizing their mechanical properties, biocompatibility, and ability to promote mesenchymal stem cell (MSC) differentiation into cardiomyocyte-like cells.

Chitosan, a naturally derived hydrophilic polymer, is recognized for its favorable mechanical strength and excellent biocompatibility, making it suitable for biomedical applications [10,11]. As shown in Table 3, the 300 kDa chitosan film exhibited significantly higher tensile strength than the 70 kDa film. This result is consistent with previous studies indicating that chitosan’s mechanical performance improves with increasing molecular weight [25,26]. To better reflect application conditions, swelling behavior was evaluated by surface area expansion rather than by conventional weight-based methods. This approach is particularly relevant for SPE applications, where maintaining structural geometry under hydrated conditions is crucial. Excessive lateral swelling can deform or delaminate printed electrodes, which may impair both structural integrity and electrochemical function. The low swelling ratio observed in the 300 kDa film (Table 3) indicates excellent aqueous stability. These characteristics make it particularly suitable for SPE fabrication in wet, physiologically dynamic environments. Similar conclusions have been drawn in previous literature, indicating that higher-molecular-weight chitosan tends to exhibit superior mechanical properties, including enhanced tensile strength and stiffness, due to increased chain length and stronger intermolecular interactions [9]. These characteristics promote structural cohesion and limit water uptake, which aligns with our findings that 300 kDa chitosan films demonstrate greater dimensional stability under hydrated conditions. This supports the use of high-molecular-weight chitosan as a reliable substrate material for SPEs operating in moist and mechanically active environments. Nevertheless, it is worth noting that the solvent system used for film preparation may also influence the polymer’s structural integrity. Acetic acid, while commonly employed for dissolving chitosan due to its biocompatibility and low toxicity, has been reported to disrupt crystalline regions and diminish inherent hydrophobicity, potentially compromising long-term material stability [27]. In our case, the 300 kDa chitosan films retained favorable mechanical and biological performance, suggesting that such effects were limited under the current conditions. However, future studies may explore alternative solvents such as ionic liquids or weaker acid systems to better preserve chitosan’s native properties while ensuring processability.

Prior to electrochemical analysis, the adhesion of screen-printed electrodes to chitosan films was evaluated to ensure reliability. Silver ink adhered well to both 70 kDa and 300 kDa chitosan films under wet conditions. This is consistent with previous findings showing strong adhesion between silver ink and polymeric substrates [28]. In contrast, carbon ink showed poor adhesion to chitosan films, although it performed well on synthetic substrates such as polycarbonate. This result highlights the importance of selecting ink–substrate combinations that are compatible within biodegradable systems. Silver ink’s strong adhesion and high conductivity make it the preferred choice for both in vitro and in vivo applications [29]. Cyclic voltammetry was used to evaluate the electrochemical performance of the silver-based SPEs (Figure 6). The electrodes exhibited well-defined redox peaks and efficient electron transfer, consistent with prior studies on silver electrodes [30]. The electrochemical responses of SPEs printed on 70 kDa and 300 kDa films were comparable, suggesting that molecular weight has minimal influence on redox behavior. The 300 kDa film, therefore, offers an ideal combination of mechanical robustness and electrochemical compatibility for reliable device fabrication. While structural preservation was observed histologically, the current study did not include functional cardiac assessments such as echocardiography or pressure–volume analysis. Future studies will integrate these techniques to provide direct evidence of myocardial recovery and validate the therapeutic potential of the patch in larger-scale models.

To further evaluate the suitability of the chitosan–SPE patch for cardiac applications, its in vivo biocompatibility was assessed by implanting it onto the infarcted myocardium of rats. The patch elicited no significant immune response or foreign body reaction, which is in agreement with previous research on the use of chitosan in biomedical applications such as wound healing and tissue repair [31]. Additionally, the chitosan–SPE patch maintained adequate toughness and flexibility during surgical handling, withstanding suturing without damage to the printed electrodes. These findings confirm the patch’s suitability for application on the dynamic cardiac surface.

In the myocardial infarction (MI) model, untreated control animals exhibited typical adverse remodeling, including myocardial thinning and infarct expansion. In contrast, animals treated with the chitosan–SPE patch displayed reduced infarct size and a thicker ventricular wall. Partial alignment of myocardial fibers was also observed, suggesting improved tissue organization. These outcomes indicate that the chitosan–SPE patch provided temporary mechanical support to the damaged myocardium, thereby reducing structural deterioration. Although complete myocardial regeneration was not achieved, the patch demonstrated therapeutic effects in the absence of exogenous cells or growth factors. This finding is consistent with prior studies showing that acellular biomaterial patches can promote partial cardiac repair [10]. However, the limited extent of cardiomyocyte regeneration and alignment implies that the patch alone may not be sufficient to fully guide functional myocardial integration.

To further investigate the regenerative capacity of the chitosan–SPE patch, in vitro studies were conducted to evaluate its effect on MSC differentiation. The combination of electrical stimulation and 5-azacytidine (5-Aza) treatment significantly increased the expression of early cardiac markers such as GATA-4 and β-MHC, followed by the later appearance of structural proteins such as Troponin I. This time-dependent expression pattern reflects a typical progression of cardiac lineage commitment, which is consistent with earlier findings [32,33,34]. The results highlight the synergistic effects of electrical and chemical stimulation in enhancing cardiomyogenic differentiation and maturation. These observations emphasize the value of combining biophysical and biochemical cues to improve in vitro cardiac differentiation outcomes [12,35].

Among all experimental groups, the chitosan–SPE patch combined with electrical stimulation and 5-Aza treatment (SPE-ES-5Aza) produced the highest levels of cardiac-specific gene expression. This result demonstrates the ability of the patch to integrate multiple stimuli that direct stem cell fate. These findings are consistent with the literature showing that 5-Aza facilitates cardiomyogenic differentiation, while electrical stimulation promotes the maturation of cardiomyocyte-like cells [36]. The presence of a conductive microenvironment appears to enhance cellular responsiveness, which is critical for improving the efficacy of tissue repair.

To enable precise control of electrical stimulation during MSC differentiation, a customized cell culture platform was developed. The setup, shown in Figure 1, allowed real-time stimulation within a culture chamber, facilitating controlled investigation of electrical effects on stem cell fate. This platform not only advances understanding of biophysical influences on cell differentiation but also demonstrates potential for future translational applications in cardiac tissue engineering.

In summary, this work demonstrates that chitosan–SPE patches are promising candidates for myocardial repair and cell-guided modulation. Future research should incorporate regenerative enhancements, including stem cell seeding, growth factor delivery, or gene editing, to promote stronger tissue integration and functional recovery. Additionally, evaluating long-term performance in large animal models will be crucial for clinical translation. The integration of mechanical stability, biocompatibility, and dual-mode stimulation in chitosan–SPE patches positions them as valuable platforms for advancing cardiac tissue engineering.

## 5. Conclusions

This study demonstrates the promising potential of chitosan-based screen-printed electrodes (SPEs) for cardiac tissue engineering. The chitosan–SPE patches exhibited favorable mechanical strength, dimensional stability, biocompatibility, and electrochemical performance, making them well suited for both in vitro and in vivo applications. In cell-based studies, the platform effectively promoted the differentiation of mesenchymal stem cells into cardiomyocyte-like cells through synergistic electrical and chemical stimulation. Upon in vivo implantation, the chitosan–SPE patch attenuated adverse myocardial remodeling and preserved tissue architecture, demonstrating its feasibility as a biodegradable cardiac scaffold. Building on these results, future research may explore the integration of regenerative cues, such as stem cells or growth factors, to further support myocardial repair and functional recovery. These findings highlight the potential of chitosan-based SPEs as a versatile platform for developing next-generation cardiac patches with improved therapeutic outcomes.

## Figures and Tables

**Table 1:** Experimental groups and treatment conditions for the in vitro study.

Group Name	Treatment Description
**SPE**	MSCs cultured on chitosan–SPEs only
**SPE-5Aza**	MSCs + SPEs + 5-azacytidine
**SPE-ES**	MSCs + SPEs + electrical stimulation
**SPE-ES-5Aza**	MSCs + SPEs + 5-azacytidine + electrical stimulation

**Table 2:** Real-time PCR primer sequences.

	Sequences (5′-3′)	Accession No.	Product
Arbp	Forward: GTACCATTGAAATCCTGAGCGATGTG Reverse: GATGCTGCCATTGTCAAACACCTG	NM_022402.2	130 bp
GATA4	Forward: GTCCCAGACATTCAGTACTGTGTCCG Reverse: GTGACAGGAGATGGATAGCCTTGTGG	NM_144730.1	99 bp
β-MHC	Forward: CACAGATGCCGCCATGATGG Reverse: CGATCTGCTCTGCCTCGTCCAG	NM_017240.1	134 bp
Troponin I	Forward: CCATGATGCAGGCACTACTGGG Reverse: GGTTTTCCTTCTCAATGTCCTCCTTC	NM_017144.1	99 bp

**Table 3:** Mechanical properties of chitosan films with different molecular weights. Tensile stress and Young’s modulus were calculated from force displacement data.

M.W.	ΔL (mm)	Tensile Strain	Tensile Stress (kPa)	Young’s Modulus (kPa)
70 kDa	21.86	0.55 ± 0.11	56.27 ± 4.95	105.39 ± 23.99
300 kDa	21.94	0.55 ± 0.05	83.65 ± 11.34	152.57 ± 18.53

## Data Availability

The original contributions presented in this study are included in the article/Supplementary Material. Further inquiries can be directed to the corresponding author.

## Supplementary Materials

The following supporting information can be downloaded at: https://www.mdpi.com/article/10.3390/polym17152088/s1, Figure S1: Surface morphology of chitosan films observed under scanning electron microscopy (SEM); Figure S2: Schematic illustration of the surgical procedure for myocardial infarction (MI) model establishment and subsequent implantation of the chitosan-based cardiac patch in rats; Figure S3: Gross morphological appearance of rat hearts before and after induction of myocardial infarction.
