# Supplementary material for: Fabrication and Evaluation of Screen-Printed Electrodes on Chitosan Films for Cardiac Patch Applications with In Vitro and In Vivo Evaluation
Source: Polymers (Basel). 2025 Jul 30;17(15):2088. doi: 10.3390/polym17152088 (PMC12349067; doi:10.3390/polym17152088)
Supplement: Supplementary file 1 [file polymers-17-02088-s001.zip › polymers-3741484-supplementary.pdf]

Supplementary Materials

For

Fabrication and Evaluation of Screen-Printed Electrodes on Chitosan Films for Cardiac Patch Applications with In Vitro and In Vivo Evaluation

Yu-Hsin Lin [1†], Yong-Ji Chen [2†], Jen-Tsai Liu[3†], Ching-Shu Yen [2], Yi-Zhen Lin [2], Xiu-Wei Zhou [2], Shu-Ying Chen [2], Jhe-Lun Hu [2], Chi-Hsiang Wu [2], Ching-Jung Chen [4*], Pei-Leun Kang [1*], and Shwu-Jen Chang [2*]

1 Department of Cardiovascular Surgery, Pingtung Veterans General Hospital, Pingtung City, 900053, Taiwan.

2 Department of Biomedical Engineering, I-Shou University, Kaohsiung City 824005, Taiwan

3 Research Center for Materials Science and Opti-Electronic Technology, College of Materials Science and Opto-Electronic Technology, University of Chinese Academy of Sciences, Beijing, 100049, China

4 Research Center for Materials Science and Opti-Electronic Technology, School of Optoelectronics, University of Chinese Academy of Sciences, Beijing, 100049, China

* Correspondence: cjchen@ucas.ac.cn (C.-J.C.); p290@ptvgh.gov.tw (P.-L. K.); sjchang@isu.edu.tw (S.-J.C.)

† These authors contribute equally to this work.

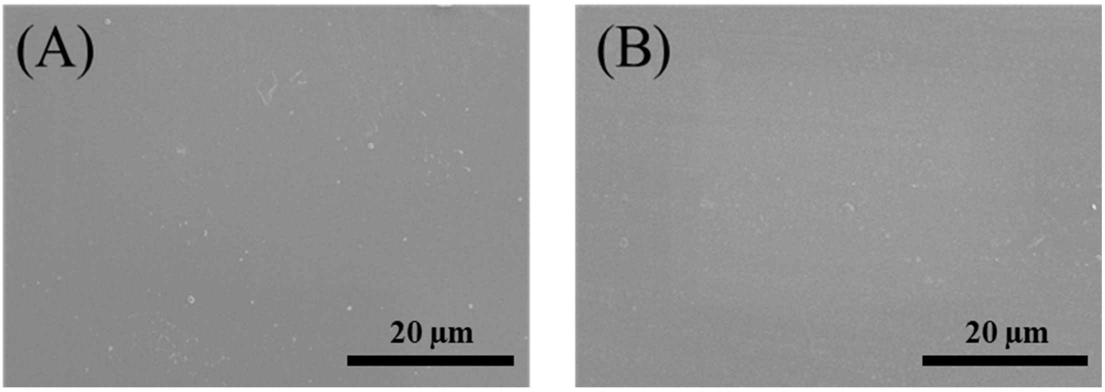

Figure S1. Surface morphology of chitosan films observed under scanning electron microscopy (SEM). (A) SEM image of the surface of 300 kDa chitosan film. (B) SEM image of the surface of 70 kDa chitosan film. Magnification: 2000×.

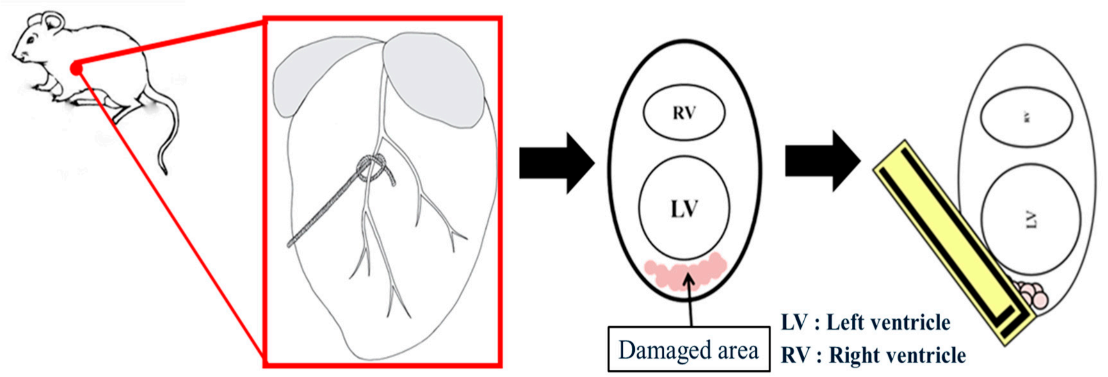

Figure S2. Schematic illustration of the surgical procedure for myocardial infarction (MI) model establishment and subsequent implantation of the chitosan-based cardiac patch in rats.

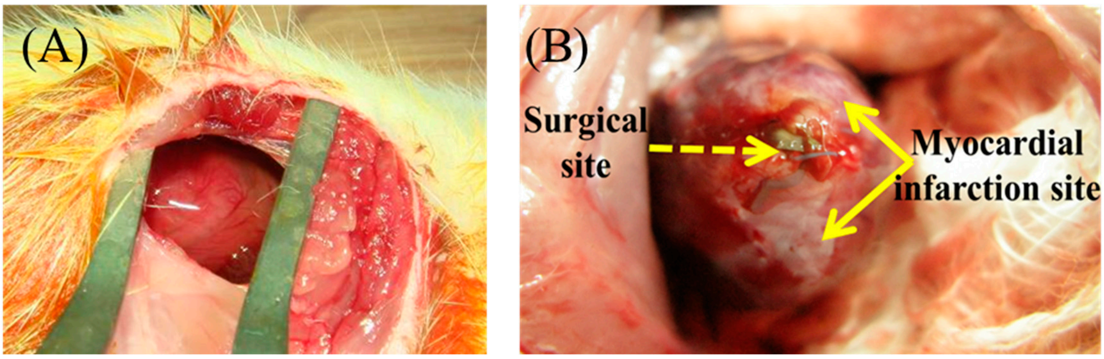

Figure S3. Gross Morphological Appearance of Rat Hearts Before and After Induction of Myocardial Infarction.

(A) Representative image of a healthy rat heart showing uniform red myocardial tissue without visible lesions.

(B) Three weeks post-LAD ligation, the infarcted heart exhibits a distinct pale white region, indicating the formation of fibrotic tissue and confirming successful myocardial infarction induction.
